# The Population Genomics of Sunflowers and Genomic Determinants of Protein Evolution Revealed by RNAseq

**DOI:** 10.3390/biology1030575

**Published:** 2012-10-25

**Authors:** Sébastien Renaut, Christopher J. Grassa, Brook T. Moyers, Nolan C. Kane, Loren H. Rieseberg

**Affiliations:** 1 Department of Botany, University of British Columbia, 3529-6270 University Boulevard, Vancouver, BC V6T 1Z4, Canada; 2 Department of Biology, Indiana University, 1001 East Third Street, Bloomington, IN 47405, USA

**Keywords:** RNAseq, gene expression, sequence divergence, *Helianthus*, selection, transcriptome, McDonald-Kreitman test

## Abstract

Few studies have investigated the causes of evolutionary rate variation among plant nuclear genes, especially in recently diverged species still capable of hybridizing in the wild. The recent advent of Next Generation Sequencing (NGS) permits investigation of genome wide rates of protein evolution and the role of selection in generating and maintaining divergence. Here, we use individual whole-transcriptome sequencing (RNAseq) to refine our understanding of the population genomics of wild species of sunflowers (*Helianthus* spp.) and the factors that affect rates of protein evolution. We aligned 35 GB of transcriptome sequencing data and identified 433,257 polymorphic sites (SNPs) in a reference transcriptome comprising 16,312 genes. Using SNP markers, we identified strong population clustering largely corresponding to the three species analyzed here (*Helianthus annuus*, *H. petiolaris*, *H. debilis*), with one distinct early generation hybrid. Then, we calculated the proportions of adaptive substitution fixed by selection (*alpha*) and identified gene ontology categories with elevated values of *alpha*. The “response to biotic stimulus” category had the highest mean alpha across the three interspecific comparisons, implying that natural selection imposed by other organisms plays an important role in driving protein evolution in wild sunflowers. Finally, we examined the relationship between protein evolution (*d*_N_/*d*_S_ ratio) and several genomic factors predicted to co-vary with protein evolution (gene expression level, divergence and specificity, genetic divergence [*F*_ST_], and nucleotide diversity *pi*). We find that variation in rates of protein divergence was correlated with gene expression level and specificity, consistent with results from a broad range of taxa and timescales. This would in turn imply that these factors govern protein evolution both at a microevolutionary and macroevolutionary timescale. Our results contribute to a general understanding of the determinants of rates of protein evolution and the impact of selection on patterns of polymorphism and divergence.

## 1. Introduction

Achieving a better understanding of the factors that shape patterns of divergence across genes, a central aim in evolutionary genetics, should become increasingly straightforward as the amount of sequencing data available grows exponentially [1,2,3]. A robust and simple way to quantify rates of gene evolution at the protein levels comes from *d*_N_/*d*_S_ratio measurements [4]. This approach quantifies selection pressures by comparing the rate of substitutions at silent sites (*d*_S_), which are presumed neutral, to the rate of substitutions at non-silent sites (*d*_N_, amino acid changes), which may experience selection. A number of genomic parameters have been shown to correlate with rates of protein coding evolution in model organisms, including gene expression level and specificity, gene length, recombination rate, and mutation rate. A general conclusion is that gene expression level, specificity and essentiality account for a substantial amount of variation in rates of protein evolution due to selective constraints imposed by these genomic parameters [1,5,6,7,8]. On the other hand, divergence in protein sequence appears to be decoupled from divergence in expression levels. While early studies of a limited number of genes [9,10] did identify a positive correlation between the rate of protein evolution and expression divergence, an absence of such correlations is more commonly reported (e.g., [11,12]).

Although significant progress has been made toward understanding the genomic factors influencing gene evolution, most of these observations have come from studies of model organisms. Also, the majority of these comparisons have been among evolutionary distant species, and the relative importance of these genomic parameters at a microevolutionary time scale is less clear (e.g., [13]). This is especially true for plant studies describing the causes of evolutionary rate variation among nuclear genes; they are almost entirely restricted to relatively divergent species in the genus *Arabidopsis* or to evolutionary distant crop species such as *Sorghum bicolor*, *Zea mays* and *Oryza sativa* [14]. As a consequence, previous studies have estimated genetic divergence in terms of nucleotide substitutions rather than *F*_ST_, since the latter will be close to one in genetically distant species.

The North American annual wild sunflowers (*Helianthus* spp.) are emerging as a model system for adaptation and speciation genomics research [15,16]. Given their well characterized evolutionary and ecological history and more recently, their genomes, sunflowers are ideal for testing predictions about the roles of gene flow, the timing of divergence, and the role of gene expression divergence in the maintenance of a plant species’ unique genome. The present study focuses on three closely related species: *Helianthus annuus*, *H. debilis* and *H. petiolaris*, which have diverged from one another less than two million years ago [16,17]. *Helianthus annuus* is widespread throughout much of the central and western United States. In southern Texas, morphologically distinct populations of *H. annuus* are described by Heiser [18] as an endemic subspecies (*H. annuus texanus*), yet these are fully interfertile with other populations of *H. annuus*. Populations of *H. annuus*, including those of ssp. *texanus*, are karyotypically divergent from *H. debilis* and *H. petiolaris* and have strong post-zygotic barriers that reduce, but do not eliminate, gene flowbetween them [18,19,20]. The three species also appear to have different ecological requirements, with *H. annuus* found mostly on moist, clay-based soils, whereas *H. debilis* and *H. petiolaris* are restricted to drier, sandy soils [21,22].

Illumina sequencing of expressed genes (RNAseq), a recently developed approach to transcriptome profiling that uses deep-sequencing technologies, has several advantages over classical microarray studies [23]. Not only does RNAseq provide more precise estimates of expression levels, but it also permits direct comparisons between gene expression and gene sequence divergence. This approach then yields a more informative and accurate view of the transcriptome at a lower cost compared with Sanger sequencing and/or microarrays. High throughput/low cost sequencing has been especially valuable for studies of non-model species by making genome sequencing, transcriptome sequencing and single nucleotide polymorphism (SNP) discovery efficient and accessible, even in the absence of a reference genome (e.g., [24]).

Here, we take advantage of next generation sequencing (RNAseq) data that was generated for *H. annuus*, *H. debilis* and *H. petiolaris* populations. Our objectives were to (i) confirm the genetic relationship among samples, then (ii) determine the effect of selection on protein coding evolution and finally (iii) investigate the effect of genomic factors which, based on previous studies, are expected to co-vary with rates of protein coding divergence. In turn, this will reveal if patterns observed in previous studies comparing more distantly related taxa also hold on shorter evolutionary timescales in recently diverged wild sunflowers.

## 2. Results and Discussion

### 2.1. Results

**Figure 1:**
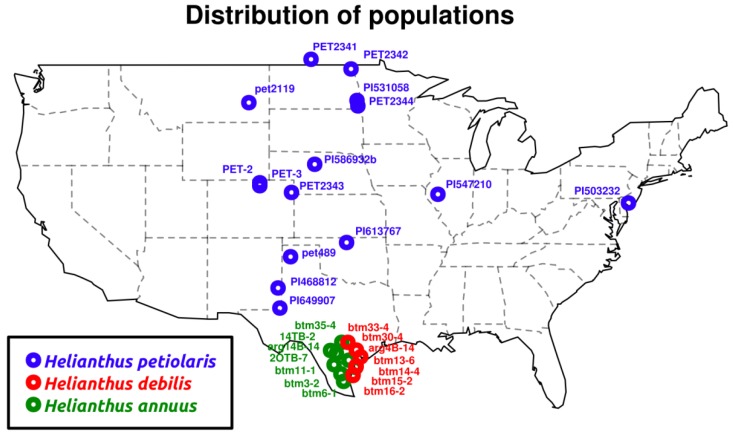
Original sample collection locations.

We generated a total of 122 GB (122 × 109 basepairs or 609 million paired-end reads) of sequencing data from RNA extracted from the 29 seedlings representing three *Helianthus* taxa: *H. annuus*, *H. debilis*, and *H. petiolaris* (Figure 1 and Table 1 and Table 2, summary of sequencing statistics). We have deposited the reference transcriptome, SNP table and raw read counts per gene in the Dryad Digital Repository [25]. Following strict quality cutoffs (see methods), we kept 67 GB of sequencing data for alignment, of which 35 GB (52%) aligned to the reference transcriptome. One *H. petiolaris* (PI586932) individual yielded less than a third of the number of reads compared the average number of reads for all other individuals. These reads were also of lower quality and therefore we discarded this sample from further analyses. Following strict quality threshold in alignments and SNP calling, we discovered 433,257 polymorphic sites in 13,412 unique contigs covering 14.5 MB, with an average of 3.0 SNPs per 100 bp (Table 1). 

**Table 1:** Summary statistics (alignments).

Total number of reference contigs = 16,312			
Total number of polymorphic contigs = 13,412			
Total number of polymorphic sites = 433,257			
**Samples**	***H. annuus***	***H. petiolaris***	***H. debilis***
Mean number of reads aligned per contig*** a***	382	798	894
Median number of reads aligned per contig*** a***	109	155	198
Mean depth per polymorphic site*** a***	75	102	107
Median depth per polymorphic site*** a***	23	32	32

***a***Note that these numbers vary mainly due to the different number of reads generated per sample (samples sequenced in duplex for *H. annuus* and triplex for *H. petiolaris* and *H. debilis*). For more results on an individual basis, see Table 2.

**Table 2:** Summary of sequencing and alignment statistics per individuals.

Species	Sample name a	Raw (GB b)	Clean c (GB)	Aligned (GB)	% aligned/total	% aligned/clean	Median coverage per contig
***H. annuus***	***14B-14***	3.58	1.38	0.72	20	52	100
***H. annuus***	***14TB-2***	3.38	1.24	0.53	16	43	70
***H. annuus***	***btm3-2***	3.48	1.38	0.69	20	50	123
***H. annuus***	***btm6-1***	3.88	1.52	0.8	21	53	127
***H. annuus***	***2OTB-7***	3.64	1.42	0.73	20	51	123
***H. annuus***	***btm11-1***	2.46	1.0	0.54	22	54	88
***H. annuus***	***btm35-4***	3.64	1.44	0.76	21	53	131
***H. debilis***	***Arg4B-14***	5.54	3.16	1.69	31	53	204
***H. debilis***	***btm30-4***	5.24	3.38	1.9	36	56	244
***H. debilis***	***btm33-4***	3.64	1.84	0.97	27	53	113
***H. debilis (praecox)***	***btm13-6***	4.86	3.12	1.62	33	52	225
***H. debilis (praecox)***	***btm14-4***	3.94	2.46	1.35	34	55	152
***H. debilis (praecox)***	***btm15-2***	6.74	3.44	1.75	26	51	281
***H. debilis (praecox)***	***btm16-2***	4.78	2.7	1.44	30	53	171
***H. petiolaris***	***pet489***	4.84	2.98	1.57	32	53	201
***H. petiolaris***	***pet2119***	5.52	3.46	1.71	31	49	275
***H. petiolaris***	***PET2341***	4.66	3.32	1.78	38	54	204
***H. petiolaris***	***PET2342***	3.88	3.06	1.67	43	55	175
***H. petiolaris***	***PET2343***	4.62	2.42	1.26	27	52	185
***H. petiolaris***	***PET2344***	4.96	2.66	1.4	28	53	166
***H. petiolaris***	***PI468812***	3.7	2.66	1.51	41	57	142
***H. petiolaris***	***PI503232***	5.24	2.52	1.29	25	51	165
***H. petiolaris***	***PI531058***	3.04	2.44	1.29	42	53	132
***H. petiolaris***	***PI547210***	3.82	1.92	1.02	27	53	120
***H. petiolaris***	***PI586932* d**	1.38	---	---	---	---	---
***H. petiolaris***	***PI613767***	5.58	3.02	1.54	28	51	180
***H. petiolaris***	***PI649907***	5.32	2.54	1.36	26	54	165
***H. petiolaris***	***PET-2***	3.36	2.38	1.3	39	55	78
***H. petiolaris***	***PET-3***	3.1	2.2	1.08	35	49	86

Note: a sample names starting with *PI* are USDA accession numbers, which can be found at [26]; b GB = 1 × 109 bases; c See methods for read cleaning procedure; d This sample had a low sequencing output and sequences were also of lower quality than all other samples. As such, it was removed from further analysis.

#### 2.1.1. Population Genetics

**Figure 2:**
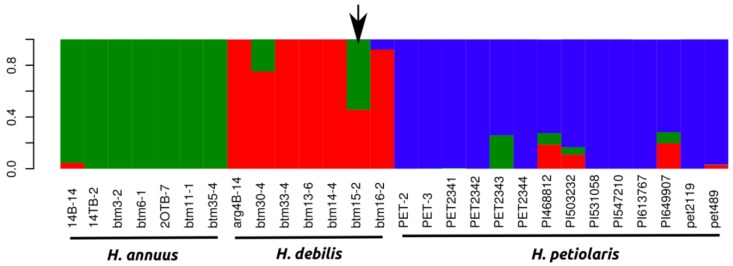
Bayesian clustering analysis of all individuals with the program structure. Individual assignment to the most probable number of clusters K (3) as inferred from the ΔK statistic (ΔK for k2 = 84.6, ΔK for k3 = 713.1 and ΔK for k4 = 12.25). Arrow indicates a putative *H. annuus* x *H. debilis* hybrid, which was removed from later analyses.

We used a random subset of 1,000 markers to conduct a Bayesian inference of population structure, which revealed a strong peak in ΔK for K = 3 (ΔK for k2 = 84.6, ΔK for k3 = 713.1 and ΔK for k4 = 12.25) largely corresponding to the species *H. annuus*, *H. petiolaris* and *H. debilis/praecox* (Figure 2). We analyzed population structure using three different sets of 1,000 random markers and obtained the same strong clustering results each time. One individual (btm15–2) clearly stood out as an early generation *H. debilis*-*H. annuus* hybrid in all runs conducted (Figure 2). We removed this individual from further analyses of genetic, protein coding and gene expression divergence.

Given that no distinct genetic clusters between *H. debilis* and *H. praecox* were identified at K = 3 (Figure 2) and the analysis of population structure with k = 4 did not reveal a clear split, all *H.praecox* individuals were subsumed within *H. debilis* for further analyses of genetic, protein coding and gene expression divergence. We also calculated the average mean *F*_ST_per gene as 0.25, 0.23 and 0.28 for *H. annuus*-*H. debilis*, *H. annuus*-*H. petiolaris*, *H. debilis*-*H. petiolaris* comparisons respectively (Table 1).

#### 2.1.2. Protein Coding Evolution and Effect of Selection

We obtained open reading frames (regions greater than 300 bp without stop codons) for 12,310 of the 16,312 reference sequences. We then used paml to calculate *d*_N _and *d*_S_in a maximum likelihood framework. Mean *d*_N_/*d*_S_ratios were 0.203, 0.203 and 0.205 for the *H. annuus-H. debilis*, *H. annuus-H. petiolaris* and *H. debilis-H. petiolaris* comparisons, respectively (Table 3). Lastly, *d*_N _and *d*_S_were strongly correlated (r2 values between *d*_N_and *d*_S_were 0.36, 0.33, 0.27 for *H. annuus-H. debilis*, *H. annuus-H. petiolaris* and *H. debilis-H. petiolaris* comparisons, respectively).

**Table 3:** Summary statistics (population genomics).

Comparison	*H. annuus-H. debilis*	*H. annuus-H. petiolaris*	*H. debilis-H. petiolaris*
Mean *F*_ST_ per gene (13,412 genes)	0.28	0.23	0.25
Median *F*_ST_ per gene (13,412 genes)	0.26	0.22	0.21
**Coding SNPs (12,310 Open Reading Frames)**			
Synonymous polymorphic SNPs (*Ps*)	147,193	162,867	174,480
Non-synonymous polymorphic SNPs (*Pn*)	104,969	116,701	126,668
Synonymous fixed (*F*_ST_ > 0.9) SNPs (*Ds*)	3,339	2,612	865
Non-synonymous fixed (*F*_ST_ > 0.9) SNPs (*Dn*)	3,581	2,935	873
*alpha* (proportion of base substitutions fixed by natural selection) and confidence intervals	0.26	0.29	0.19
	[0.22–0.31]	[0.24–0.34]	[0.11–0.29]
G-statistic (*p*-value) *	239	231	23
	(2.2e–16)	(2.2e–16)	(1.4e–6)
*d*_N_ */d*_S_	0.22	0.24	0.22

* G-test conducted as in [27].

We then tested whether the ratio of non-synonymous to synonymous fixed differences was greater than the non-synonymous to synonymous polymorphism ratio using a g-test. For all comparisons, there was a significant excess of non-synonymous fixed differences (*g*-test < 0.0001 in all cases, Table 3). The genome wide proportion of base substitutions fixed by natural selection (*alpha*) was observed to be 0.28 for *H. annuus-H. debilis*, 0.23 for *H. annuus-H. petiolaris* and 0.25 for *H. debilis-H. petiolaris*. When focusing on the most abundant GO categories (comprising at least 50 genes), we found evidence that the proportion of adaptive evolution (*alpha*) varied significantly with the class of biological processes considered. In Table 4, we present the five GO categories with the highest *alpha* value in each comparison. These GO categories comprised a number of biological processes. In only one case, however, was the same GO category (response to biotic stimulus) present in the “top five” in all three species pairs. Other GO categories with very high *alpha* values in at least one comparison included flower development, glycolysis, defense response to fungus, lipid catabolic process, and lipid metabolic process.

**Table 4:** Proportion of adaptive substitutions (*alpha* and confidence intervals) in the GO categories (n > 50 genes) undergoing the highest adaptation (values in bold represent values found in the five GO categories with the highest alpha values in each comparison respectively).

			alpha		
Description	Number of genes	Gene ontology category (GO)	*H. annuus/H. debilis*	*H. annuus/H. petiolaris*	*H. debilis/H. petiolaris*
All annotated genes	11,081 (total)		0.26 ± 0.04	0.29 ± 0.05	0.19 ± 0.09
apoptosis	84	GO:0006915	0.28 ± 0.03	0.3 ± 0.04	**0.79 ± 0.03**
defense response to fungus	63	GO:0050832	0.33 ± 0.05	0.56 ± 0.03	**1 ± 0**
flower development	94	GO:0009908	0.54 ± 0.04	**0.63 ± 0.03**	0.4 ± 0.12
glycolysis	76	GO:0006096	**0.83 ± 0.02**	**0.71 ± 0.02**	0.72 ± 0.04
GTP catabolic process	103	GO:0006184	0.55 ± 0.02	0.29 ± 0.04	−0.14 ± 0.08
ion transmembrane transport	85	GO:0034220	0.45 ± 0.03	**0.65 ± 0.03**	−0.41 ± 0.14
lipid catabolic process	130	GO:0016042	0.45 ± 0.03	0.52 ± 0.03	**0.76 ± 0.02**
lipid metabolic process	243	GO:0006629	0.45 ± 0.03	0.25 ± 0.04	**0.88 ± 0.02**
lipid transport	94	GO:0006869	**0.58 ± 0.02**	0.48 ± 0.02	−0.04 ± 0.23
mitosis	79	GO:0007067	**0.59 ± 0.02**	0.59 ± 0.04	0.64 ± 0.04
response to biotic stimulus	60	GO:0009607	**0.63 ± 0.02**	**0.8 ± 0.01**	**1 ± 0**
translational initiation	89	GO:0006413	**0.67 ± 0.03**	0.03 ± 0.06	−0.93 ± 0.14
ubiquitin-dependent protein catabolic process	105	GO:0006511	0.43 ± 0.06	**0.65 ± 0.03**	0.14 ± 0.2

#### 2.1.3. Genomic Determinants of Rates of Protein Evolution

**Figure 3:**
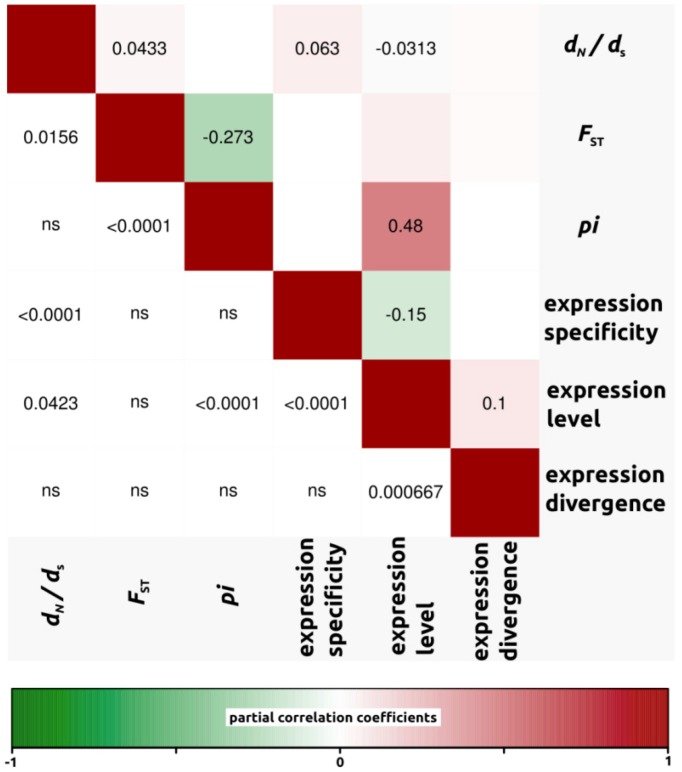
Partial correlation coefficients aiming to understand the genomic factors associated with variation in protein coding sequence. Partial correlation coefficients (above diagonal), color-coded according to sign and degree of correlation, and *p*-values (below diagonal). Average of all three comparisons (4,908, 4,161 and 4,196 ORFs for *H. annuus-H. debilis*, *H. annuus-H. petiolaris* and *H. debilis-H. petiolaris* comparisons, respectively).

**Figure 4:**
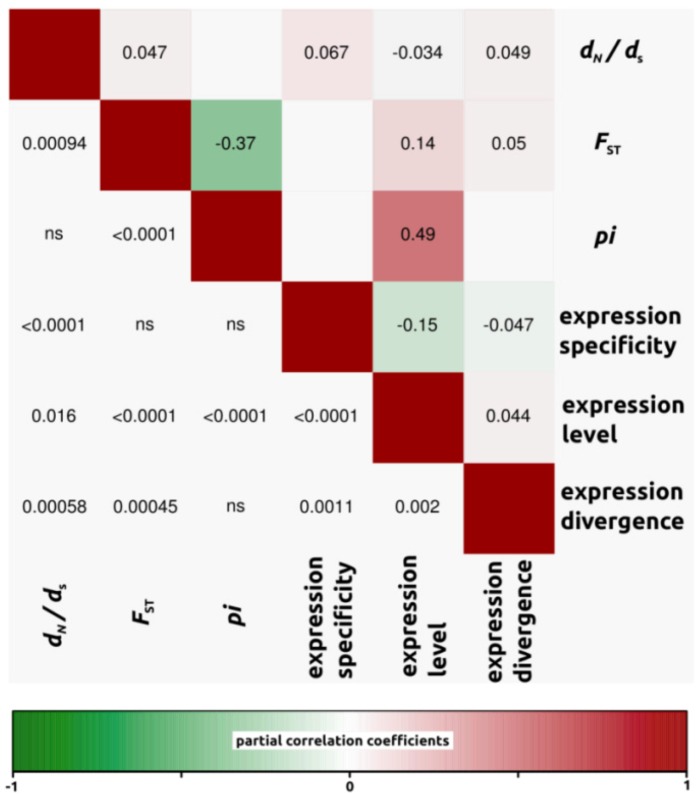
Partial correlation coefficients aiming to understand the genomic factors associated with variation in protein coding sequence. Partial correlation coefficients (above diagonal), color-coded according to sign and degree of correlation, and *p*-values (below diagonal). *H. annuus-H. debilis* comparison (4,908 ORFs).

**Figure 5:**
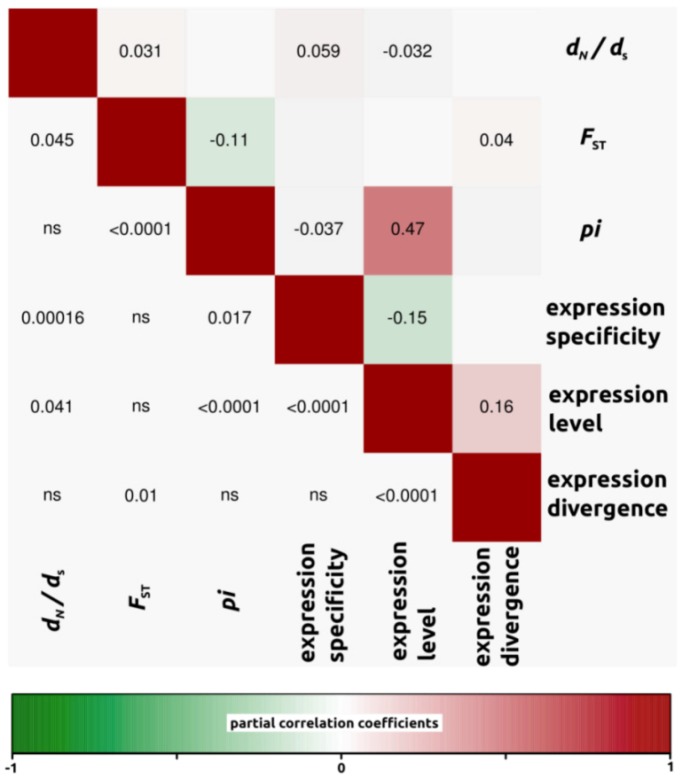
Partial correlation coefficients aiming to understand the genomic factors associated with variation in protein coding sequence. Partial correlation coefficients (above diagonal), color-coded according to sign and degree of correlation, and *p*-values (below diagonal). *H. annuus-H. petiolaris* comparison (4,161 ORFs).

**Figure 6:**
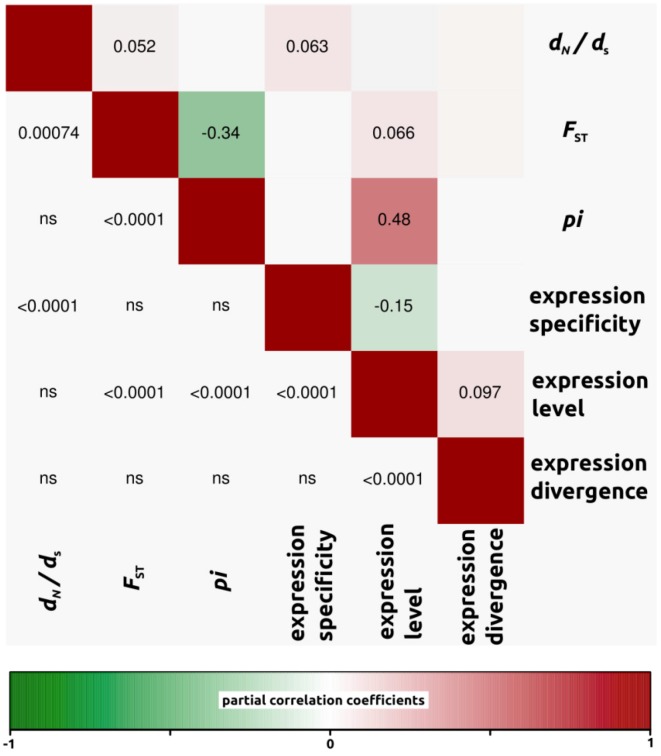
Partial correlation coefficients aiming to understand the genomic factors associated with variation in protein coding sequence. Partial correlation coefficients (above diagonal), color-coded according to sign and degree of correlation, and *p*-values (below diagonal). *H. debilis-H. petiolaris* comparison (4,196 ORFs).

We conducted partial correlation analyses among different factors which have been previously shown to correlate with variation in rates of protein evolution. First, we only analyzed *d*_N_/*d*_S_ ratios for open reading frames in which both *d*_N_ and *d*_S_ were different from zero (4,908, 4,161 and 4,196 ORFs for *H. annuus-H. debilis*, *H. annuus-H. petiolaris* and *H. debilis-H. petiolaris* comparisons, respectively). Then, we noticed that for all three interspecific comparisons, correlation coefficients were similar. Therefore, in order to simplify the interpretations, we have presented the data as a mean of the three comparisons (Figure 3, but see also Figure 4, Figure 5, Figure 6 for correlations per species pair). Rates of protein divergence (*d*_N_/*d*_S_) were negatively correlated with gene expression level (partial cor. coef. = −0.03) and positively with gene expression specificity (0.06), consistent with results from a broad range of taxa [6,8,28] and positively correlated with overall sequence divergence (*F*_ST_, partial cor. coef. = 0.04). Among other notable results predicted from previous studies, we found a strong and significant negative correlation between expression level and specificity (−0.15). Nucleotide diversity (*pi*) was strongly negatively correlated with sequence divergence (−0.27) and surprisingly, positively with expression level (partial cor. coef. = 0.48). We also found that sequence divergence (*F*_ST_) did not correlate with expression divergence.

### 2.2. Discussion

#### 2.2.1. Next-Generation Sequencing Era

Our approach using a reference transcriptome of 16,312 contigs allowed to survey a substantial fraction of all genes expressed and capture genome wide signals that would likely go undetected when using a handful of anonymous markers or a candidate gene strategy. Nearly every gene in our reference set had a least two sequences align to it and could therefore be considered expressed (15,756 out of 16,312 contigs) in the sequenced samples. The expression cut-offs commonly employed for microarrays or RT-PCR has created a perception that genes with a signal intensity below a certain ambient noise are not expressed. However, this perception probably relates more to the resolving power of the method rather than a real property of the gene; genes usually considered as silent are probably often expressed at a very low level making expression differences more subtle than originally perceived [29]. Conversely, we cannot rule out this possibility in some cases of uncertainty in sequence or alignment; for this reason genes with less than two aligned reads were removed from the data set. The fact that only about 50% of cleaned sequences aligned to our reference indicates that a significant fraction of the transcriptome remains un-assembled and/or that some reads are too divergent from the reference or represent chimeric sequences. Alternatively spliced genes and genes filtered from the reference dataset because they were inferred to represent families of close paralogs might also explain some of this missing fraction. In RNAseq studies, another source of bias can come from aligning reads from other species to a reference composed of a single species, here *H. annuus*. Fortunately, this does not seem to be a problem given the alignment parameters we chose and the fact that the same percentages (52%) of cleaned reads (Table 2) were aligned in all three species.

Given that the cultivated sunflower genome is nearing completion [30], there will soon be a more accurate reference to align sequences against. A fully sequenced genome will permit more accurate identification of gene space, alternative splicing events, untranslated 5' and 3' regions and intron/exon boundaries. In addition, we are concurrently developing reference transcriptomes for several closely related sunflower species including *H. petiolaris* and *H. debilis*. This will allow us to investigate orphan genes that are present in either *H. petiolaris* or *H. debilis* but have no detectable homolog in *H. annuus*. Such genes are rare, but may play an important role in generating species-specific evolutionary novelties [31]. Clearly, we have only begun to understand the evolutionary complexity underlying both sequence and expression divergence.

#### 2.2.2. Population Genetics

Annual species of sunflowers in the genus *Helianthus* vary greatly in their geographic distribution and evolutionary origin. Based on Bayesian clustering of nuclear markers spanning the whole genome, we found a clear distinction between *H. debilis*, *H petiolaris* and *H. annuus* individuals. However, structure often does not distinguish hierarchical sub-structuring and consequently, may only reveal the top level of population differentiation. Our study was not intended to resolve the phylogeny of *Helianthus*, as this would have needed much more extensive sampling. However, our results corroborate previous phylogenetic work [32,33] that places *H. praecox* within *H. debilis*.

As a whole, genetic divergence was surprisingly similar among all three species comparisons (Table 1) despite the fact that the divergence of *H. annuus* with *H. petiolaris* and *H. debilis* clearly precedes the *H.petiolaris*-*H. debilis* divergence [33]. Hybridization between recognized species is known to participate in shaping the evolutionary diversity of North American sunflowers [20,34,35] and probably explains the patterns observed here. H. *petiolaris* and *H. debilis* are allopatric sister species, but both hybridize with the sympatric but evolutionary more distant *H. annuus*. *H. annuus* and *H. debilis* are known to have a long history of introgression in Texas, where these individuals were collected [20,35]. Similarly, *H. petiolaris* forms numerous hybrid zones with *H. annuus* throughout their ranges [34]. As such, these more distantly related species have exchanged genes across much of the genome while remaining morphologically and ecologically distinct [16], presumably accounting for lower than expected average *F*_ST_ values.

#### 2.2.3. Protein Coding Evolution

The longest open-ended ORFs (minimum length of 300 nucleotides) were kept as the most probable translated region of the gene. SNPs within these ORFs were considered as coding sites as thus it could be assessed whether these were synonymous or non-synonymous. This approach (as compared with a blastx approach to known protein sequences from model systems) has the advantage of identifying genes that have no detectable homolog present in public databases. Such orphan genes are potentially rare, but may play an important role in generating species-specific evolutionary novelties [31].

Our results are consistent with several expectations based on previous research [14]. First, mean *d*_N_*/d*_S_ ratios were close to 0.2 for all three comparisons and few genes were identified with values greater than one, signaling strong constraint on amino acid replacements. This is consistent with the results of Yang and Gaut [28] who analyzed protein coding evolution between *Arabidopsis thaliana* and *A. lyrata* and estimated *d*_N_*/d*_S_ at 0.203. Many studies have documented a positive correlation between *d*_N_ and *d*_S_ across genes, similar to that identified here [14]. The reason for this correlation is not well understood, although it may be caused by processes such as strong purifying selection towards translational efficiency or variation in mutation rates and recombination rates along chromosomes, which would affect both *d*_N_and *d*_S_ in a similar fashion.

Based on calculations of the genome wide proportion of base substitutions fixed by natural selection (*alpha*), we conclude that a significant proportion of the most divergent SNPs represent selectively advantageous mutations: in all cases the ratio of non-synonymous to synonymous fixed differences was significantly greater than that for non-synonymous to synonymous polymorphisms (*alpha*, Table 3). By focusing on specific gene categories and examining patterns of polymorphism and divergence, we were able to provide additional evidence of adaptive evolution (Table 4). Gene categories exhibiting high *alpha* values included a number of biological processes that have previously been reported as undergoing positive selection in sunflowers, including defense response to fungus, flower development, lipid catabolic process, lipid metabolic process, and response to biotic stimulus [36,37,38,39,40]. Interestingly, we found one category, response to biotic stimulus, showing parallel levels of elevated *alpha* among species pairs. This is important given that parallel trends add stronger evidence for the role of selection in driving divergence for these particular traits [41]. Thus, natural selection imposed by other organisms appears to play an important role in driving protein evolution in wild sunflowers. However, we also acknowledge the dangers of story telling based on GO analysis and these trends should be confirmed before any strong conclusions can be reached [42].

#### 2.2.4. Genomic Determinants of Rates of Protein Evolution

To understand the underlying causes of evolutionary rate heterogeneity among genes, researchers have investigated correlations between evolutionary rates and various genomic parameters [1,8,28]. In accordance with previous studies (e.g., [28]), we found a significant negative correlation between *d*_N_/*d*_S_ ratios and expression level, with genes that are highly expressed being more constrained in terms of protein evolution. Likewise, *d*_N_/*d*_S_ ratios were positively correlated with expression specificity. Again the leading explanation for a positive correlation is that widely expressed genes are more constrained in protein sequence evolution because they are probably involved in multiple biochemical pathways and face multiple selective environments in the different tissues they are expressed in [5]. Finally, we found a strong and significant negative correlation between expression level and specificity. Highly expressed genes are usually involved in a larger number of biochemical processes and expressed in a greater number of different tissues than lowly expressed genes. Since specificity was determined based on relatively shallow (44,000 reads) sequencing, lowly expressed genes may appear to have high specificity due to the stochastic lack of sampling in some libraries. At the same time, it is likely that with enough power (*i.e.*, enough sequencing), we may find that all genes are expressed nearly everywhere (*i.e.*, expression is leaky, see [29]). Therefore specificity may eventually need to be redefined or at least measured in independent samples, as in the present study.

All the trends reported above are consistent with theory and results from a broad range of taxa [1,2,3,5,7,28,43]. Interestingly however, the correlation coefficients reported here are generally lower than those observed in previous studies. This is probably due to the more recent divergence of sunflowers compared with the more distantly related taxa studied previously. For instance, the sunflowers species analyzed here have diverged less than two million years ago [19], compared with about eight MYA in the *Arabidopsis thaliana*-*lyrata* comparisons [28], six MYA for the *Drosophila melanogaster* group (but with much faster generation times than sunflowers [1,44], and about 75 MYA in the case of the mouse and human lineages [5]. As recently pointed out in a study quantifying protein coding evolution broadly across the Asteraceae family [13], it appears that, at least to some degree, macroevolution looks very much like “repeated rounds of microevolution” [45]. Given that our results are also qualitatively similar to previous studies conducted at a more ancient timescale, we argue that macroevolutionary trends are governed by the same principles of microevolution. Yet, the importance of the genomic factors discussed above is possibly reduced relative to the idiosyncratic effects of drift and local variation in selection pressures when analyzed over shorter timescales [13].

Unlike previous studies, we also observed several new and sometimes unexpected associations. For one, we found that *d*_N_/*d*_S_ ratios were positively correlated with overall genetic divergence (*F*_ST_). Given that high values of *d*_N_/*d*_S_ and *F*_ST _can result from positive selection, this association could be expected. Among other noticeable results, we did not find a significant correlation between genetic divergence (measured as *F*_ST_, or *d*_N_/*d*_S_ ratios) and expression divergence. After observing no correlation in yeast between *d*_N_ and expression divergence, Tirosh and Barkai [11] suggested that certain genes are more sensitive to changes in coding sequences, whereas others are more sensitive to changes in non coding regions regulating expression. Consequently, an absence of correlation between patterns of expression and genetic divergence, as we identified in the present study, suggests a decoupling of evolutionary forces shaping expression and genetic divergence.

Unexpectedly, nucleotide diversity (*pi*) was strongly correlated with expression level in *Helianthus*. With RNAseq, the confidence with which nucleotide variants are identified is a function of coverage, which itself represents expression. As a consequence, greater coverage (expression) increases the probability of identifying both alleles at heterozygous loci and may inflate the total number of variants [46]. Analyzing only the subset of genes with *d*_N_ and *d*_S_ greater than 0 should ensure that most low coverage genes are removed (mean expression value for all genes in dataset = 365, compared with mean expression of genes used in partial correlation analysis = 665). Using an even more stringent criterion on the minimum expression of genes (for example, minimum expression threshold of 100 instead of now 14) does slightly reduce the correlation between *pi* and expression level but does not substantially affect the other partial correlations reported in Figure 3. Thus, our conclusions hold when using more stringent filtering procedures. Lastly, nucleotide diversity (*pi*) was also strongly negatively correlated with *F*_ST_. This is not unexpected and likely pertains to the definition of *F*_ST_ itself ([heterozygosity between-heterozygosity within]/heterozygosity between).

Essentially all previous studies looking at the genomic determinants of sequence evolution have employed one dataset comprised of sequenced genes, a separate dataset for calculating gene expression (*i.e.*, microarray data often measured in different individuals or even populations) and another dataset to identify genomic context (*i.e.*, an annotated genome). In addition, previous studies have mainly estimated genetic divergence in terms of nucleotide substitutions between distant species, rather than *F*_ST_, since the latter will be close to one in genetically distant species. Because of the independence between the expression and sequence data sets in these previous studies, the apparent ascertainment bias detected in the present study is avoided. Thus, while RNAseq increases the precision of expression analyses, and ensures that the same pairs of genes are compared for sequence and expression analyses, the approach is not without drawbacks.

## 3. Experimental Section

### 3.1. Sample Preparation, Sequencing and Reference

We collected achenes (single seeded fruits) from seven *Helianthus annuus* and seven *H. debilis* individuals in Texas. Four of the *H. debilis* individuals were initially labeled as *H. praecox*, yet these clustered with *H. debilis* individuals genetically (see below). As molecular phylogenetic studies of *Helianthus* also place *H. praecox* within *H. debilis* [32,33], we follow an early taxonomy [47] that subsumes *H. praecox* into *H. debilis*. In addition, we acquired twelve *H. petiolaris* accessions spanning the distribution range of the species, either from USDA collections or from previous sampling efforts (Figure 1).

We germinated all achenes at the University of British Columbia (Vancouver, Canada) and grew them for approximately three weeks under growth chambers or similar greenhouse conditions (12 hours of daylight at 22 degrees). Then we harvested all aboveground biomass, flash froze it in liquid nitrogen and kept it at −80 degrees. For each individual, we extracted RNA from young leaves and stems using a modified TRIzol Reagent protocol (Invitrogen, Carlsbad, CA, USA). We quantified the RNA samples using a NanoDrop (Thermo Fisher Scientific, Waltham, MA, USA) and verified their quality on agarose gels. We stored the total RNA in pure water. Finally, samples were retrotranscribed using a custom Invitrogen (now Life Technologies, Carlsbad, CA, USA) cDNA kit, nearly identical to the SuperScript^®^ Double-Stranded cDNA Synthesis Kit. After shearing by sonication, cDNA fragments 190 to 210 basepairs in length were isolated and PCR-amplified to generate the sequencing library, following standard Illumina (San Diego, CA, USA) Genome Analyzer paired-end library protocols. These were sequenced on a GAII Illumina platform (paired end sequencing, 2 × 100 bp reads) at the Genome Sciences Centre (Vancouver, Canada) and base calling was done as part of the standard pipelines at the Genome Sciences Centre sequencing facility. Samples were multiplexed (three samples per lane for *H. annuus* and two per lane for all others).

Our reference Expressed Sequence Tags (EST) set consists of 16,312 contiguous sequences (contigs). It was assembled from available Sanger and 454 EST sequences generated for *H. annuus* (both wild and cultivated individuals spanning the whole distribution of the species) and from several different tissue types [48]. Individual genotypes were first assembled with mira version 3.0 [49], using the flags “accurate,est,denovo,454”. All mira contigs and singletons were reassembled further using the program cap3 at 94% identity [50] as in [24]. Contigs found in at least two different genotypes and with total length greater than 500 bp were kept for the final merged assembly. Based on comparisons to genetic map data [51], 783 contigs contain sequences that map to multiple locations and were removed, as they likely represent families of close paralogs that cannot be resolved. Of the 16,312 remaining sequences, 8,226 map to single locations on genetic maps [51] and appear to be single copy, while the remainder was not assessed. In addition to the mapped and unmapped nuclear loci, this reference set also contains the whole chloroplast genome, which spans nearly 127 KB. This assembly has therefore gone through strict quality trimming and should represent the majority of genes expressed in young seedlings. 

### 3.2. Alignments

We first cleaned the raw sequencing files (fastq sequencing files in Illumina 1.3+ format) to remove any low quality reads and potential contaminating vector sequences. To ensure high quality of data, we discarded reads where one or more position had a phred quality score below 3 (in either paired end). We then aligned the reads against the reference transcriptome using bwa (aln and sampe command, default parameters except for read trimming parameter (-q) set at 20 to ensure high quality alignments [52]). Following alignments, we used the Indel Realigner from the genome analysis toolkit (gatk [53]) to correct alignment errors near indel regions. We used samtools [54] to extract the base-pair information at each site for each individual (pileup command, 17 million sites). Note that one individual (PI586932) was not included in the analysis given low sequencing yield (see results section). We parsed this output (base-pair information at each site for each individual) based on several criteria. First, sites with a read depth of less than three were called as missing. Then, if that site passed this first filter, the genotype was called as heterozygous only if the minor allele was represented by more than two reads and the minor allele frequency was at least 10%. At this point, we concatenated information from all individuals for all sites. From this dataset, we filtered on a per site basis, in order to keep only sites for which no more than 20% of all individuals had missing data, expected heterozygosity (*H*e) was greater than 0.2 and observed heterozygosity (*H*o) was smaller than 0.6. Polymorphic sites with values above the latter threshold likely represent paralogous sequence variants instead of true SNPs. Conversely, polymorphic sites with values below the former threshold either represent rare alleles, which possess little information for the sake of differentiating individuals, or sequencing errors (unless very high coverage is attained). Nevertheless, we are aware that our analysis likely contains a small fraction of false positives due to alignment and/or sequencing errors. However, given the large amount of data, high overall coverage, strict quality thresholds cut-offs and visual inspection of random subsets of alignments (several tens of kilobases), we expect the data to be more than sufficient for the analysis we report here. This is evident when the same trends are observed when conducting replicate analysis using small random subsamples (for example in the structure runs, see results).

### 3.3. Population Genetics

To ensure that individuals clustered according to their described species, we assessed nuclear genetic structure through Bayesian model-based clustering in structure v.2.3 [55]. structure determines the most likely number of differentiated clusters (K) represented by the sample and assigns the sampled genotypes to the inferred clusters. Using a random subset of 1,000 markers, we estimated the log likelihood of the data, given different numbers of genetic clusters K, using an admixture model with correlated allele frequencies, without sampling locations as priors and with all other parameters as defaults. For each k value of 1 through 6, we ran ten replicates (50,000 burn-in cycles, 100,000 MCMC iterations), from which we calculated ΔK following [56]. We used the program structure harvester [57] to identify the number of populations K with the best support.

### 3.4. Protein Coding Evolution and Effect of Selection

We identified open reading frames (ORF) in our reference transcriptome using the program getorf in emboss (European Molecular Biology Open Software Suite [58]). We kept the longest open-ended ORF (minimum length of 300 nucleotides) as the most probable translated region of the gene. We used paml (Phylogenetic Analysis by Maximum Likelihood [59], which accounts for codon usage bias and GC content, to calculate the rate of non synonymous mutations scaled by the rate of synonymous mutations (*d*_N_/*d*_S_) in a maximum likelihood framework (run-mode = 0, CodonFreq = 2, model = 2). For *d*_N_/*d*_S_ calculations, we first identified the consensus (major) allele for each site for each species and then annotated these sites into a single consensus sequence per gene per species. We discarded genes where either *d*_N_or *d*_S_ equalled zero, keeping only those *d*_N_/*d*_S_ ratios that could be calculated with confidence.

We first assessed the effect of selection on protein coding evolution globally. We tested if the ratio of non-synonymous to synonymous fixed differences was greater than the ratio of non-synonymous to synonymous polymorphisms using a g-test as in [27]. As an extension of this approach, the average proportion of amino-acid substitutions driven by positive selection (*alpha*) can be estimated using equation 3 in [60]

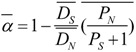

where all averages are across genes and *P*_S_, *D*_S _, *P*_N_ and *D*_N_ are the number of synonymous polymorphisms, synonymous substitutions, nonsynonymous polymorphisms and nonsynonymous substitutions, respectively. Confidence intervals of *alpha* were obtained through bootstraps (1,000 bootstraps) by randomly selecting genes with replacement as described in [60]. In this case, substitutions (*D*) were considered as SNPs with an *F*_ST_value greater or equal to 0.9.

Another way of extracting ecologically relevant information is through the characterization of functional categories that are over-represented within certain subsets of genes [61]. We therefore examined patterns of adaptive protein evolution for distinct gene ontology categories as in [8]. We first compared genes using blastx and then employed the best hits to assign gene ontology (GO) annotation terms based on the uniprot database [62]. We calculated *alpha* for GO categories comprised of at least fifty genes and identified categories with the highest *alpha* levels (the top five for each comparison respectively).

### 3.5. Genomic Determinants of Protein Evolution

To assess what genomic factors are correlated with rates of protein evolution, we estimated partial correlation coefficients in the programming language r (v.2.13.0. [63], package ppcor.r  [64]). We assessed the degree of partial correlation between protein divergence (log(*d*_N_/*d*_S_)), genetic divergence (mean *F*_ST_per gene), genetic diversity (*pi*, calculated in sites [65], expression specificity, log(expression level), and the absolute value of log(expression species 1/expression species 2). For genetic diversity (*pi*), we also considered using the Watterson estimator *theta* given that this parameter may have a different bias than *pi* [66]. Both estimators (*pi* and *Watterson*) were strongly correlated (r2 = 0.96) and partial correlation coefficient results were largely the same for both. As such, we only report values of *pi*. We calculated *F*_ST_values for each SNP (according to [67] using the r package hierfstat [68] and then averaged it per gene.

We measured gene expression tissue specificity using a dataset of publicly available Sanger sequences for *H. annuus* developed by the Compositae Genome Project [48]. Briefly, these libraries were constructed using eleven different tissues from two genotypes (callus, roots, disk and ray flowers, pre-fertilized flowers, developing kernel, chemically treated seedlings, environmentally stressed roots, environmentally stressed shoots, germinating seeds, environmentally stressed flowers, and hulls, see [48] for details). These libraries were Sanger sequenced to generate a total of 44,000 barcoded reads. We demultiplexed and compared all reads (blastx, *e-*value < 1e-10) against our reference transcriptome of 16,312 genes. We defined expression specificity (range 0–11) as the total number of libraries (11) minus the number of libraries where a gene scored at least one hit as in [7].

For gene expression calculations (level and divergence), we first normalized the number of reads aligned per gene per individual by the median expression of each library. Normalization via the common approach of Reads per Kilobase per Million Mapped Reads (RPKM) can be strongly biased by a relatively small proportion of highly expressed gene and we therefore decided against using it [69]. Given that we were also interested in interpreting differences in expression between genes, we normalized all expression data by gene length*.* Then, we used the r package deseq [70] to calculate expression divergence per gene, as the absolute mean value of expression for species 1 divided by expression for species 2 on a base two logarithmic scale.

## 4. Conclusions

As the cost of sequencing continues to drop, RNAseq is expected to replace microarrays for many applications that involve determining the structure and dynamics of the transcriptome [23]. While likely to revolutionize our understanding of transcriptome evolution, especially in non-model systems, a possible caveat is that sources of bias in coverage and therefore gene expression estimates are not yet fully understood. For example, it appears that base composition, library preparation and transcript length may all impact gene expression estimates [23]. As reviewed by [3], many challenges remain when dealing with next generation sequence data. In addition, more work is necessary to generalise the relationship between the evolution of promoter sequences, protein coding regions and gene expression in plants [14]. Our study provides a significant advance in our understanding of molecular evolution. It reveals several general trends that appear to be robust to variables such as timing of divergence or experimental growth conditions, given that they have been reported across a broad range of taxa. At the same time, a meta-analysis of studies conducted under different environmental conditions, developmental stages, and species may reveal novel insights into plant molecular evolution that are not apparent when studying only a single condition. With the increased availability of high-throughput sequencing datasets, such large-scale studies are becoming increasingly feasible.

## References

[1] Larracuente A.M., Sackton T.B., Greenberg A.J., Wong A., Singh N.D., Sturgill D.S., Zhang Y., Oliver B., Clark A.G. (2008). Evolution of protein-coding genes in *Drosophila*. Trends Genet..

[2] Gilad Y., Pritchard J., Thornton K., Thornton K. (2009). Characterizing natural variation using next-generation sequencing technologies. Trends Genet..

[3] Stapley J., Reger J., Feulner P.G.D., Smadja C., Galindo J., Ekblom R., Bennison C., Ball A.D., Beclerman A.P., Slate J. (2010). Adaptation genomics: The next generation. Trends Ecol. Evol..

[4] Kryazhimskiy S., Plotkin J.B. (2008). The Population Genetics of dN/dS. PLoS Genet..

[5] Duret L., Mouchiroud D. (2000). Determinants of Substitution Rates in Mammalian Genes: Expression Pattern Affects Selection Intensity but Not Mutation Rate. Mol. Biol. Evol..

[6] Pal C., Papp B., Lercher M.J. (2006). An integrated view of protein evolution. Nat. Rev. Genet..

[7] Ingvarsson P.K. (2007). Gene expression and protein length influence codon usage and rates of sequence evolution in *Populus tremula*. Mol. Biol. Evol..

[8] Slotte T., Bataillon T., Hansen T.T., Onge K.S., Wright S.I., Schierup M.H. (2011). Genomic determinants of protein evolution and polymorphism in *Arabidopsis*. Genome Biol. Evol..

[9] Nuzhdin S., Wayne M., Harmon K., McIntyre L. (2004). Common pattern of evolution of gene expression level and protein sequence in *Drosophila*. Mol. Biol. Evol..

[10] Khaitovich P. (2005). Parallel Patterns of Evolution in the Genomes and Transcriptomes of Humans and Chimpanzees. Science.

[11] Tirosh I., Barkai N. (2008). Evolution of gene sequence and gene expression are not correlated in yeast. Trends Genet..

[12] Jeukens J., Renaut S., St-Cyr J., Nolte A.W., Bernatchez L. (2010). The transcriptomics of sympatric dwarf and normal lake whitefish (*Coregonus*
*clupeaformis* spp., Salmonidae) divergence as revealed by next-generation sequencing. Mol. Ecol..

[13] Kane N.C., Barker M.S., Zhan S.H., Rieseberg L.H. (2011). Molecular Evolution across the Asteraceae: Micro- and Macroevolutionary Processes. Mol. Biol. Evol..

[14] Gaut B., Yang L., Takuno S., Eguiarte L.E. (2011). The Patterns and Causes of Variation in Plant Nucleotide Substitution Rates. Annu. Rev. Ecol. Evol. Syst..

[15] Rieseberg L., Willis J. (2007). Plant Speciation. Science.

[16] Kane N.C., King M.G., Barker M.S., Raduski A., Karrenberg S., Yatabe Y., Knapp S., Rieseberg L.H. (2009). Comparative genomic and population genetic analyses indicate highly porous genomes and high levels of gene flow between divergent *Helianthus* species. Evolution.

[17] Sambatti J., Strasburg J.L., Ortiz-Barrientos D., Baack E.J., Rieseberg L.H. (2012). Reconciling extremely strong barriers with high levels of gene exchange in annual sunflowers. Evolution.

[18] Heiser C.B. (1951). Hybridization in the Annual Sunflowers-*Helianthus-annuus* X *H-argophyllus*. Am. Nat..

[19] Strasburg J.L., Rieseberg L.H. (2008). Molecular demographic history of the annual sunflowers *Helianthus annuus* and *H. petiolaris*-Large effective population sizes and rates of long-term gene flow. Evolution.

[20] Scascitelli M., Whitney K.D., Randell R.A., King M., Buerkle C.A., Rieseberg L.H. (2010). Genome scan of hybridizing sunflowers from Texas (*Helianthus*
*annuus* and *H debilis*) reveals asymmetric patterns of introgression and small islands of genomic differentiation. Mol. Ecol..

[21] Heiser C.B. (1947). Hybridization between the sunflower species *Helianthus*
*annuus* and H. petiolaris. petiolaris. Evolution.

[22] Heiser C.B. (1951). Hybridization in the annual sunflowers: *Helianthus annuus* × *H. debilis* var. cucumerifolius. Evolution.

[23] Wang Z., Gerstein M., Snyder M. (2009). RNA-Seq: A revolutionary tool for transcriptomics. Nat. Rev. Genet..

[24] Lai Z., Kane N.C., Kozik A., Hodgins K.A., Dlugosch K.M., Barker M.S., Matvienko M., Yu Q., Turner K.G., Pearl S.A. (2012). Genomics of Compositae weeds: EST libraries, microarrays, and evidence of introgression. Am. J. Bot..

[25] Dryad. http://dx.doi.org/10.5061/dryad.rs4k0/.

[26] United States Department of Agriculture. http://www.arsgrin.gov/npgs/acc/acc_queries.html/.

[27] McDonald J.H., Kreitman M. (1991). Adaptive protein evolution at the adh locus in *Drosophila*. Nature.

[28] Yang L., Gaut B.S. (2011). Factors that Contribute to Variation in Evolutionary Rate among Arabidopsis Genes. Mol. Biol. Evol..

[29] Ptitsyn A. (2008). Stochastic Resonance Reveals “Pilot Light” Expression in Mammalian Genes. PLoS One.

[30] Kane N.C., Gill N., King M.G., Bowers J.E., Berges H., Gouzy J., Bachlava E., Langlade N.B., Lai Z., Stewar M. (2011). Progress towards a reference genome for sunflower. Botany.

[31] Khalturin K., Hemmrich G., Fraune S., Augustin R. (2009). More than just orphans: Are taxonomically-restricted genes important in evolution?. Trends Genet..

[32] Rieseberg L.H. (1991). Homoploid reticulate evolution in *Helianthus* (Asteraceae): Evidence from ribosomal genes. Am. J. Bot..

[33] Timme R.E., Simpson B.B., Linder C.R. (2007). High-resolution phylogeny for *Helianthus* (Asteraceae) using the 18S-26S ribosomal DNA external transcribed spacer. Am. J. Bot..

[34] Rieseberg L., Whitton J., Gardner K. (1999). Hybrid zones and the genetic architecture of a barrier to gene flow between two sunflower species. Genetics.

[35] Heiser C.B. (1954). Variation and Subspeciation in the Common Sunflower, *Helianthus Annuus*. Am. Midl. Nat..

[36] Linder C.R. (2000). Adaptive Evolution of Seed Oils in Plants: Accounting for the Biogeographic Distribution of Saturated and Unsaturated Fatty Acids in Seed Oils. Am. Nat..

[37] Whitney K.D., Randell R.A., Rieseberg L.H. (2006). Adaptive Introgression of Herbivore Resistance Traits in the Weedy Sunflower Helianthus annuus. Am. Nat..

[38] Chapman M.A., Leebens-Mack J.H., Burke J.M. (2008). Positive Selection and expression divergence following gene duplication in the sunflower cycloidea gene family. Mol. Biol. Evol..

[39] Blackman B.K., Rasmussen D.A., Strasburg J.L., Raduski A.R., Burke J.M., Knapp S.J., Michaels S.D., Rieseberg L.H. (2011). Contributions of Flowering Time Genes to Sunflower Domestication and Improvement. Genetics.

[40] Chapman M.A., Tang S., Draeger D., Nambeesan S., Shaffer H., Barb J.G., Knapp S.J., Burke J.M. (2012). Genetic Analysis of Floral Symmetry in Van Gogh’s Sunflowers Reveals Independent Recruitment of CYCLOIDEA Genes in the Asteraceae. PLoS Genet..

[41] Schluter D., Nagel L.M. (1995). Parallel Speciation by Natural Selection. Am. Nat..

[42] Pavlidis P., Jensen J.D., Stephan W., Stamatakis A. (2012). A Critical Assessment of Storytelling: Gene Ontology Categories and the Importance of Validating Genomic Scans. Mol. Biol. Evol..

[43] Slotte T., Foxe J.P., Hazzouri K.M., Wright S.I. (2010). Genome-Wide Evidence for Efficient Positive and Purifying Selection in *Capsella grandiflora*, a Plant Species with a Large Effective Population Size. Mol. Biol. Evol..

[44] Russo C.A., Takezaki N., Nei M. (1995). Molecular phylogeny and divergence times of drosophilid species. Mol. Biol. Evol..

[45] Erwin D.H. (2000). Macroevolution is more than repeated rounds of microevolution. Evol. Dev..

[46] Gilad Y., Rifkin S., Pritchard J. (2008). Revealing the architecture of gene regulation: The promise of eQTL studies. Trends Genet..

[47] Heiser C.B. (1956). Biosystematics of *Helianthus debilis*. Madrono.

[48] Heesacker A., Kishore V.K., Gao W., Tang S., Kolkman J.M., Gingle A., Matvienko M., Kozik A., Michelmore R.M., LaI Z. (2008). SSRs and INDELs mined from the sunflower EST database: Abundance, polymorphisms, and cross-taxa utility. Theor. Appl. Genet..

[49] Chevreux B., Pfisterer T., Drescher B., Driesel A., Müller W.E.G., Wetter T., Suhai S. (2004). Using the miraEST assembler for reliable and automated mRNA transcript assembly and SNP detection in sequenced ESTs. Genome Res..

[50] Huang X., Madan A. (1999). CAP3: A DNA sequence assembly program. Genome Res..

[51] Bowers J.E., Bachlava E., Brunick R.L., Rieseberg L.H., Knapp S.J., Burke J.M. (2012). Development of a 10,000 Locus Genetic Map of the Sunflower Genome Based on Multiple Crosses. G3 Genes Genomes Genet..

[52] Li H., Durbin R. (2009). Fast and accurate short read alignment with Burrows-Wheeler transform. Bioinformatics.

[53] McKenna A., Hannal M., Banksl E., Sivchenko A., Cibulskisl K., Kernytsky A., Garimella K., Altshuler D., Gabriell S., Daly M. (2010). The Genome Analysis Toolkit: A MapReduce framework for analyzing next-generation DNA sequencing data. Genome Res..

[54] Li H., Handsaker B., Wysoker A., Fennell T., Ruan N., Homer N., Marth G., Abecasis G., Durbin R. (2009). 1000 Genome Project Data Processing Subgroup7. The Sequence Alignment/Map format and SAMtools. Bioinformatics.

[55] Pritchard J., Stephens M., Donnelly P. (2000). Inference of population structure using multilocus genotype data. Genetics.

[56] Evanno G., Regnaut S., Goudet J. (2005). Detecting the number of clusters of individuals using the software structure: A simulation study. Mol. Ecol..

[57] Earl D.A., von Holdt B.M. (2011). STRUCTURE HARVESTER: A website and program for visualizing STRUCTURE output and implementing the Evanno method. Conserv. Genet. Resour..

[58] Rice P., Longden I., Bleasby A. (2000). EMBOSS: The European molecular biology open software suite. Trends Genet..

[59] Yang Z. (0724). PAML 4: Phylogenetic analysis by maximum likelihood. Mol. Biol. Evol. 20.

[60] Smith N.G.C., Eyre-Walker A. (2002). Adaptive protein evolution in *Drosophila*. Nature.

[61] Ashburner M., Ball C.A., Blake J.A., Botstei D., Butler H., Cherry J.M., Davis A.P., Dolinski K., Dwight S.S., Eppig J.T. (2000). Gene Ontology: Tool for the unification of biology. Nat. Genet..

[62] UniProt. http://www.ebi.ac.uk/uniprot/.

[63] R Development Core Team (2012). R: A Language and Environment for Statistical Computing.

[64] Kim S.H., Yi S.V. (2007). Understanding relationship between sequence and functional evolution in yeast proteins. Genetica.

[65] SITES (Hey Lab Distributed Software). http://genfaculty.rutgers.edu/hey/%E2%80%A8software#SITES/.

[66] Watterson G.A. (1975). Theoretical Population Biology-On the number of segregating sites in genetical models without recombination. Theoret. Popul. Biol..

[67] Weir B. (1996). Genetic Data Analysis II.

[68] Goudet J. (2005). HIERFSTAT, a package for R to compute and test hierarchical F-statistics. Mol. Ecol. Notes.

[69] Bullard J.H., Purdom E., Hansen K.D., Dudoit S. (2010). Evaluation of statistical methods for normalization and differential expression in mRNA-Seq experiments. BMC Bioinformatics.

[70] Anders S., Huber W. (2010). Differential expression analysis for sequence count data. Genome Biol..

